# Cutaneous squamous cell carcinoma metastatic to parotid - analysis of prognostic factors and treatment outcome

**DOI:** 10.1186/1477-7819-10-117

**Published:** 2012-06-25

**Authors:** Robin Yeong Hong Goh, Ron Bova, Gerald B Fogarty

**Affiliations:** 1 Faculty of Medicine, University of New South Wales, Botany Street, Sydney, NSW, 2052, Australia; 2 Department of Head and Neck Surgery, St Vincent’s Hospital, 390 Victoria Street, Darlinghurst, NSW, 2010, Australia; 3 Genesis Cancer Care, St Vincent’s Clinic, 438 Victoria Street, Darlinghurst, NSW, 2010, Australia; 4 Radiation Oncology, Mater Hospital, 25 Rocklands Rd, Crows Nest, NSW, 2065, Australia

**Keywords:** Audit, Head and neck, Immune suppression, Metastasis, Neck lymph nodes, Parotidectomy, Radiotherapy, Skin cancer

## Abstract

**Background:**

Cutaneous squamous cell carcinoma (cSCC) comprises 20% of all skin cancer of the head and neck. A minority will metastasize to regional parotid lymph nodes. This study evaluates the St Vincent’s Hospital, Sydney experience between 1996 and 2006.

**Methods:**

A retrospective review was performed of patients who were evaluated in our multidisciplinary head and neck clinic with metastatic cSCC to parotid, and all treatment and pathologic details were reviewed. Statistical analysis, including univariate and multivariate analyses, were performed using Cox proportional hazards regression mode, overall and disease-specific survival were estimated by the Kaplan-Meier method.

**Results:**

Sixty-seven patients were identified. Some 90 % were male, and with a mean age of 72.8 years. One died on the first postoperative day. The remaining 66 patients received radiotherapy. For these 66 patients, the two-year and five-year overall survival rate was 0.83 and 0.72, respectively. The two-year and five-year disease-free survival rate was 0.91 and 0.83 respectively. Overall survival was only significantly correlated to the extent of parotidectomy (superficial versus total; *P* = 0.0256). Margin status was available in 59 patients. The only parameter that significantly correlated with disease-free survival was margin status (close/negative versus positive *P* = 0.0348). Other parameters of immune suppression, perineural invasion, extra capsular extension, degree of tumour differentiation, number of positive nodes, extent of neck dissection and radiotherapy dosage delivered did not confer prognostic significance.

**Conclusions:**

This study confirmed the association of adverse prognostic implication of positive margins on disease-free survival. Immune compromise was not a significant factor in this small group. Further studies are warranted in this population.

## Background

The most common malignancy in the human person is skin cancer. [1]. Australia has the highest incidence of skin cancer in the world [2]. Cutaneous squamous cell carcinoma (cSCC) incidence in Australia is rising. From 1985 to 2002, age-standardised incidence rates of cSCC had increased by 133% [2]. The mortality is also increasing [3]. Some 20% of all skin cancer of the head and neck are cSCC [4]. Only a minority of facial and scalp cSCC will metastasise to regional parotid lymph nodes. It is an Australian phenomenon that the most common malignant tumour of the parotid gland is metastatic cSCC [5]. These patients suffer increased morbidity and even mortality if locoregional control is not achieved at first presentation

Definitive treatment of metastatic cSCC to the parotid is a therapeutic challenge requiring multidisciplinary input and multimodality therapy. The purpose of this study was to evaluate our experience in treating metastatic cSCC to the parotid gland in patients presenting to our multidisciplinary head and neck clinic.

Our institutional management policy for this disease is as follows. Parotidectomy with appropriate neck dissection is considered the standard of care for patients with metastatic cutaneous SCC to the parotid gland in our institution. The extent of parotidectomy is based on preoperative clinical and radiologic assessment and follows the definitions of neck dissection defined by Robbins *et al*. [6]. Superficial parotidectomy with facial nerve conservation is appropriate for the majority of patients unless facial nerve infiltration is identified preoperatively or tumour infiltration into the facial nerve is identified at the time of surgery, in which case, total parotidectomy with facial nerve resection is considered. Similarly, if adjacent soft tissue structures such as preauricular skin or external auditory canal are involved clinically or radiologically, then extended resection encompassing the surrounding soft tissues with appropriate local or flap reconstruction is considered. Adjuvant radiotherapy to the parotid bed and ipsilateral neck starting within six weeks of operation is then given. The surgical bed is planned for treatment to 60 to 66 Gy, the neck to 45 to 56 Gy, all in 1.8 to 2 Gy fractions at five fractions per week. Treatment is discontinued if there is significant radiation toxicity. Concurrent chemotherapy was not used routinely in this patient population at this time.

## Methods

A retrospective review of patients with metastatic cSCC to the parotid gland who were treated with curative intent at St. Vincent's Hospital during a 10-year period (1996 to 2006) was performed. Research was carried out in compliance with the Helsinki Declaration and this project was approved by the St Vincent’s Hospital Human Research Ethics Committee approval number 08/108. All patients were evaluated in a multidisciplinary head and neck clinic and all clinical and pathological data was prospectively recorded. Data collected included: age, sex, immune status, primary skin tumour site if available, nodal involvement, margin status, extracapsular extension, perineural invasion, extent of surgery performed and radiotherapy details. Immune-compromised patients included patients with intercurrent haematological malignancies, lymphoma and transplant recipients on long-term immunosuppressive treatment.

Statistical analysis was performed using STATA version 9 (Stata Corp., College Station, TX, USA). Univariate and multivariate analyses were performed using Cox proportional hazards regression mode and overall survival (OS) and disease-specific survival (DSS) were estimated by the Kaplan-Meier method. The OS period was calculated from parotidectomy to date of death or last follow-up. The DSS period was calculated from parotidectomy to date of death from disease or last follow-up.

Multivariate analysis of factors influencing OS and DSS were performed on selected explanatory variables by means of a Cox’s proportional hazards regression. A step-wise approach was used to select independent significant covariates. The variables were analysed via a nominal categorization. The probabilities of entry and removal from the multivariate model were set at 0.10 and 0.10, respectively. Prognostic factors that showed significant *P* value of less than 0.1 in univariate analysis were investigated in the multivariate analyses. These were immune suppression, margin status of parotid, parotidectomy and O’Brien P stage [5].

## Results

### Patient characteristics

Sixty-seven patients that fulfilled the eligibility criteria for this study and their details are summarised in Table 1. Some 90% of the patients were male and the mean age of the patients was 72.8 years. Forty-one (61%) patients presented with metastatic cSCC to the parotid only while 26 (39%) patients presented with metastatic cSCC to both parotid and ipsilateral cervical lymph nodes. Eight patients (12%) were immune-compromised at the time of surgery. Three patients had non-Hodgkin’s lymphoma and five patients had chronic lymphocytic leukemia. Eleven patients had a history of primary lesion that was thought responsible for the parotid nodes.

**Table 1:** Patient characteristics

	**All**	**Patients with cancer spread to parotid only**	**Patients with cancer spread to parotid and neck**
N	67	41 (61.2%)	26 (38.8%)
Sex			
Female	7 (10.4%)	2 (4.9%)	5 (19.2%)
Male	60 (89.6%)	39 (95.1%)	21 (80.8%)
Age in years (mean ± SD)	72.8 ± 11.5	72.9 ± 11.4	72.7 ± 12.0
Immunosuppression			
Immune-compromised	8 (12.1%)	5 (12.2%)	3 (12.0%)
Non-immune-compromised	58 (87.9%)	36 (87.8%)	22(88.0%)

### Treatment characteristics

All patients underwent parotidectomy. Fifty-three patients had superficial parotidectomy performed while fourteen patients underwent total parotidectomy. Fifty-four patients had an ipsilateral neck dissection performed. Of these twenty-eight had radical neck dissection, thirteen had modified radical neck dissection, twelve had selective neck dissection and one patient had an extended radical neck dissection.

One patient died on the first postoperative day. This patient was excluded from the survival analysis. The remaining sixty-six patients all received planned postoperative adjuvant radiotherapy. All 66 patients received radiotherapy to the parotid bed, and 65 to the ipsilateral neck. The average dose of radiotherapy to the parotid and upper neck was 53.9 Gy (SD: 5.6 Gy), while the lower neck received an average dose of 43.6 Gy (SD: 19.8 Gy). Three received platinum-based concurrent chemotherapy.

### Pathologic characteristics

Sixty patients had a complete histopathology report from the parotidectomy available for analysis. The average number of parotid lymph nodes removed was 4.5 while the average number of positive parotid nodes removed was 1.7. The largest parotid node removed was 7 cm with 50% of positive nodes measuring greater than 2 cm. Nine patients (15%) had histological evidence of perineural invasion while thirty-five (57%) patients displayed extra capsular extension in the parotidectomy specimen.

Parotidectomy specimens were also assessed for margin status. Margin status was available in 59 patients and was classified as positive, negative or close (if < 0.5 mm). Twenty-one patients (35%) had positive margins, twenty-three (38%) were negative and fifteen patients (25%) had close margins.

### Overall survival outcomes and associations

**Figure 1:**
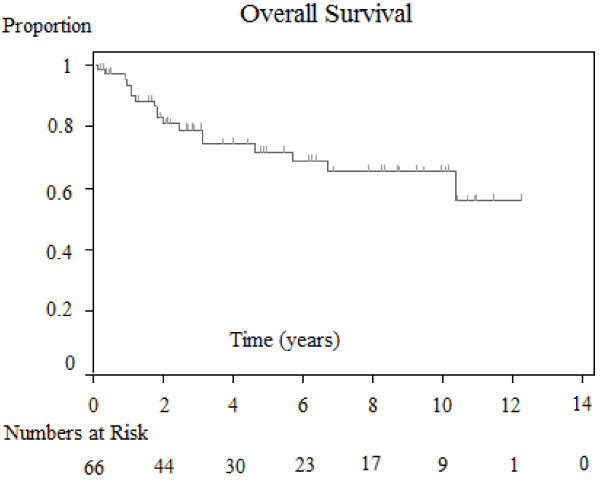
**Kaplan-Meier estimated overall survival for all patients**.

**Figure 2:**
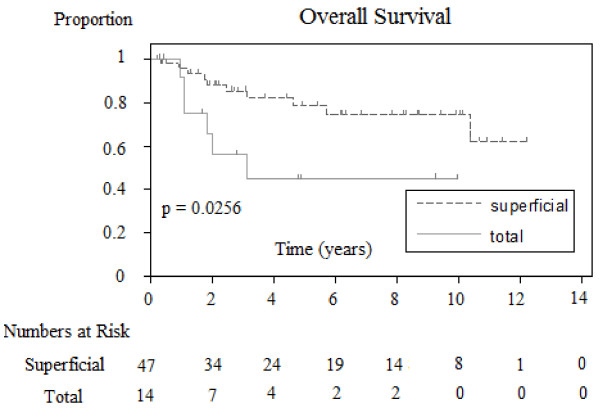
**Kaplan-Meier estimated disease-specific survival for all patients**.

For the 66 patients, the two-year and five-year overall survival rate was 0.83 and 0.72, respectively. (See Figure 1Additional file 1). Overall survival was only significantly correlated with the extent of parotidectomy (superficial versus total; *P* = 0.0256). Patients that required total parotidectomy had reduced survival compared to those that had superficial parotidectomy performed (See Figure 2). O’Brien staging [5] for overall survival was almost significant for P1 versus P2 + P3 (*P* = 0.0531). Parameters of immune suppression, perineural invasion, extracapsular extension, degree of tumour differentiation, number of positive nodes, extent of neck dissection and radiotherapy dosage delivered did not confer prognostic significance.

### Disease-specific survival outcomes and associations

**Figure 3:**
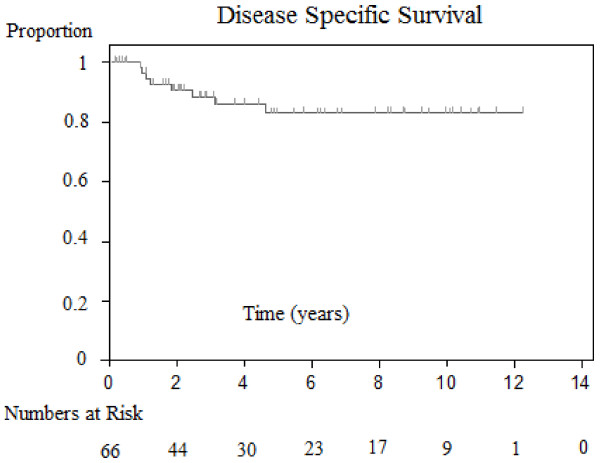
Comparison of Kaplan-Meier estimated overall survivals between patients with different extent of parotidectomy.

**Figure 4:**
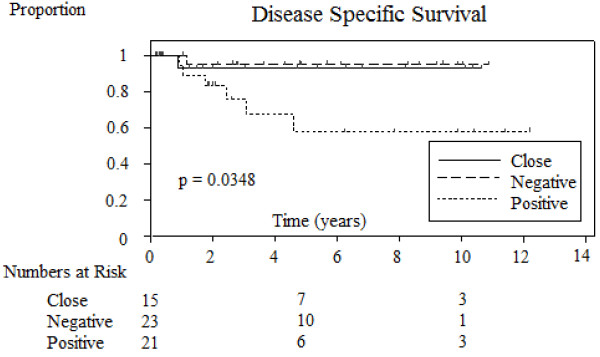
**Comparison of Kaplan-Meier estimated disease-specific survival between patients with negative, positive and close parotid margin**.

The two-year and five-year disease-free survival rate was 0.91 and 0.83 respectively. (See Figure 3, Additional file 2). The only parameter that significantly correlated with disease-free survival was margin status (Close/negative versus positive *P* = 0.0348). Patients with close or negative margins had improved two-year and five-year survival compared to patients with positive margins (See Figure 4). Parameters of immune suppression, perineural invasion, extracapsular extension, degree of tumour differentiation, number of positive nodes, O’Brien staging, extent of neck dissection and radiotherapy dosage delivered did not confer prognostic significance.

## Discussion

Metastatic cSCC to the parotid gland is a relatively common clinical presentation faced by Australian head and neck surgeons and radiation oncologists. Unfortunately, there are no randomised control trials to guide treatment recommendations, and our current management guidelines are based on similar retrospective studies from other Australian institutions.

The survival analysis showed that this cohort had similar survival rates to other studies. Table 2 compares the results of our studies to other institutions. However, the two- and five-year disease-free survival rates of 91% and 83% respectively are slightly higher than other reported series [5,7-11]. This may be because all of our analyzed patients were treated with surgery with clearance of at least all macroscopic disease, and also radiotherapy to parotid and all but one to the ipsilateral neck. The other studies included patients treated with just radiotherapy or just surgery.

**Table 2:** Comparison to other similar studies

**Series**	**Duration**	**Number**	**% Treated with surgery and adjuvant radiotherapy**	**Survival at 5 years**
O’Brien *et al*. [5]	1990–2002	87	86	63% DSS
Ch’ng *et al*. [7]	1990–2004	67	67	44% CSS
				54% DSS
				79% DFS
Chua *et al*. [8]	1980–1997	52	100	65% CSS
Palme *et al*. [9]	1987–1999	126	89	68% DSS
Dona *et al*. [10]	1983–2000	74	100	72% CSS
Veness *et al*. [11]	1980–2000	167	87	73% DFS
Goh *et al*. (current study)	1996–2006	66	100	72% OS
				83% DSS

*CSS*, cause-specific survival; *DFS*, disease-free survival; *DSS*, disease-specific survival; *OS*, overall survival.

Analysis of prognostic variables showed that patients that underwent total parotidectomy had a significantly worse outcome than patients requiring superficial parotidectomy (superficial versus total; *P* = 0.0256). This would seem logical as patients requiring total parotidectomy had more advanced locoregional disease. This study has also confirmed the adverse prognostic implication of positive margins on disease-free survival (Close/negative versus positive *P* = 0.0348). The five-year disease-free survival of patients with negative parotid margins was 88% compared with 41% for patients with positive parotid margins. This once again highlights the importance of attempting to achieve clear surgical margins. The adverse effect of positive parotid margins in patients with metastatic cSCC has also been identified in other studies [5,10]. Careful preoperative planning with appropriate imaging such as CT and/or MRI scan is recommended when tumour extension outside the parotid gland is suspected. Patients can then be appropriately counseled about the possible need for adjacent soft tissue resection and arrangements for appropriate reconstruction can be made.

The significant effect of immune compromise in this group of patients has been validated in other studies [12-14]. These patients have significantly worse overall survival than immune-competent patients. Interestingly, when we compared disease-specific survival between these two patient populations no significant survival difference was found.

The role of elective neck dissection also remains controversial. Several Australian studies have demonstrated that approximately one third of patients with metastatic cSCC to the parotid gland with clinically negative necks will have occult metastases identified following neck dissection [15,16]. Selective neck dissection is a relatively straightforward procedure associated with relatively limited morbidity, particularly when combined with parotidectomy. We routinely perform elective supraomohyoid neck dissection in conjunction with parotidectomy. There are, as yet, no randomised control trials comparing adjuvant radiotherapy to elective neck dissection in the treatment of the clinically negative neck in this disease.

## Conclusions

Cutaneous squamous cell carcinoma metastatic to the parotid is a challenging condition requiring multidisciplinary assessment and multimodality therapy. This retrospective review performed on 67 patients at St Vincent’s Hospital, Sydney, between 1996 and 2006 confirmed the adverse prognostic implication of positive margins on disease-free survival. Immune compromise was not a significant factor in this small group.

## Abbreviations

cSCC, cutaneous squamous cell carcinoma; CT, computed tomography scan; DSS, disease-specific survival; Gy, gray; MRI, magnetic resonance imaging; NMSC, non-melanoma skin cancer; OS, overall survival; SD, standard deviation.

## Supplementary Material

Additional file 1: **A table showing overall survival.** (DOC 305 kb) Click here for file

Additional file 2: **A table showing disease-specific survival.** (DOC 109 kb) Click here for file
