# Supplementary material for: Cutaneous squamous cell carcinoma metastatic to parotid - analysis of prognostic factors and treatment outcome
Source: World J Surg Oncol. 2012 Jun 25;10:117. doi: 10.1186/1477-7819-10-117 (PMC3422189; doi:10.1186/1477-7819-10-117)
Supplement: Additional file 1: — A table showing overall survival. (DOC 305 kb) [file 1477-7819-10-117-S1.doc]

**Table 2** – Overall Survival

|  | *No of patients*  *(No of events)* | *2-year survival rate* | *5-year survival rate* | *Median survival (months)* | *HR* | *Log-rank test P value* |
| --- | --- | --- | --- | --- | --- | --- |
| **Primary tumour size** | | | | | | |
| <20 mm | 6 (1) | 1.00 | 0.80 | NR |  |  |
| ≥20 mm | 5 (2) | 0.67 | 0 | 56.5 | 4.69 (0.42, 52.53) | 0.1686 |
| **Patients with metastasis at different sites** | | | | | | |
| Parotid only | 40 (9) | 0.86 | 0.72 | NR |  |  |
| Both parotid and neck | 26 (9) | 0.77 | 0.70 | 126.5 | 1.65 (0.65, 4.16) | 0.2862 |
| **Margin status of parotid (secondary tumour)** | | | | | | |
| *Patients with metastasis at Parotid only, and both parotid and neck* | | | | | | |
| Positive vs negative vs. close |  |  |  |  |  | **0.0101** |
| Negative | 23(3) | 0.90 | 0.90 | NR |  |  |
| Positive | 21(11) | 0.74 | 0.41 | 68.1 | **4.57 (1.27, 16.51)** |  |
| Close | 15(2) | 0.84 | 0.84 | NR | 1.04 (0.17, 6.25) |  |
| Positive vs. no positive |  |  |  |  |  |  |
| No positive margins | 38 (5) | 0.88 | 0.88 | NR |  |  |
| Positive margins | 21 (11) | 0.74 | 0.41 | 38.1 | **4.50 (1.55, 3.02)** | **0.0024** |
| Negative vs. Close |  |  |  |  |  |  |
| Negative | 23 (3) | 0.90 | 0.90 | NR |  |  |
| Close | 15 (2) | 0.84 | 0.84 | NR | 1.03 (0.17, 6.16) | 0.9753 |
| Broad front vs. focally |  |  |  |  |  |  |
| Broad front | 9 (4) | 0.75 | 0.47 | 56.5 |  |  |
| Focally | 12 (7) | 0.73 | 0.36 | 38.0 | 1.32 (0.37, 4.69) | 0.6691 |
| **Margin status of neck (secondary tumour)** | | | | | | |
| *Patients with metastasis both parotid and neck* | | | | | | |
| Positive vs. no positive |  |  |  |  |  |  |
| No positive margins | 18 (6) | 0.73 | 0.73 | 126.5 |  |  |
| Positive margins | 5 (2) | 0.75 | 0.38 | 56.5 | 0.96 (0.18, 4.95) | 0.9566 |
| Broad front vs. focally |  |  |  |  |  |  |
| Broad front | 1 (1) | 1.00 | 0 | NR |  |  |
| Focally | 4 (1) | 0.67 | 0.67 | NR | 0.58 (0 .03, 10.25) | 0.7055 |
| **Perineural Invasion** | | | | | | |
| No invasion | 51 (14) | 0.83 | 0.72 | NR |  |  |
| Invasion | 9 (4) | 0.71 | 0.57 | 126.5 | 1.61 (0.53, 4.91) | 0.4001 |
| **Extracapsular extension** | | | | | | |
| No extension | 26 (6) | 0.78 | 0.71 | NR |  |  |
| Extension | 34 (12) | 0.84 | 0.69 | 126.5 | 1.27 (0.47, 3.40) | 0.6339 |

|  | *No of patients (No of events)* | *2-year survival rate* | *5-year survival rate* | *Median survival (months)* | *HR* | *Log-rank test P value* | |
| --- | --- | --- | --- | --- | --- | --- | --- |
| **Differentiation of secondary metastasis** | | | | | | | |
| Poor | 33 (8) | 0.83 | 0.62 | NR |  | |  |
| Moderate | 27 (10) | 0.78 | 0.70 | 126.5 | 1.27 (0.49, 3.24) | |  |
| Well | 1 (0) | NA | NA | NR | - | | 0.6914 |
| **No of parotid node** | | | | | | | |
| *≤1 node vs. >1 node* |  |  |  |  |  | |  |
| 0 or 1 parotid node | 45 (10) | 0.84 | 0.79 | NR |  | |  |
| >1 parotid node | 20 (7) | 0.83 | 0.61 | 126.5 | 1.55 (0.59, 4.10) | | 0.3693 |
| *1 node vs. >1 node* |  |  |  |  |  | |  |
| 1 parotid node | 45 (10) | 0.84 | 0.79 | NR |  | |  |
| >1 parotid node | 20 (7) | 0.83 | 0.61 | 126.5 | 1.55 (0.59, 4.10) | | 0.3693 |
| **O’Brien Staging** | | | | | | | |
| *P stage ( P1 vs. P2 vs. P3)* | | | | | | | |
| P1 | 35 (5) | 0.86 | 0.82 | NR |  | |  |
| P2 | 26 (10) | 0.83 | 0.65 | 126.5 | 2.64 (0.90, 7.74) | |  |
| P3 | 2 (1) | 0.50 | 0.50 | 13.2 | 4.09 (0.47, 35.37) | | 0.1345 |
| *P stage ( P1 vs. P2+ P3)* | | | | | | | |
| P1 | 35 (5) | 0.86 | 0.82 | NR |  | |  |
| P2+P3 | 28 (11) | 0.80 | 0.64 | 126.5 | 2.73 (0.95, 7.86) | | 0.0531 |
| *N stage (N0 vs. N1 vs. N2)* | | | | | | | |
| N0 | 40 (9) | 0.86 | 0.72 | NR |  | |  |
| N1 | 7 (3) | 0.71 | 0.71 | 81.6 | 1.94 (0.52, 7.25) | |  |
| N2 | 19 (6) | 0.79 | 0.71 | 126.5 | 1.53 (0.54, 4.31) | | 0.5258 |
| **AJCC staging** | | | | | | | |
| *N1 vs. N2 vs. N3* |  |  |  |  |  | |  |
| N1 | 22 (4) | 0.85 | 0.79 | NR |  | |  |
| N2 | 38 (12) | 0.82 | 0.70 | 126.5 | 1.88 (0.60, 5.83) | |  |
| N3 | 1 (0) | NA | NA | NR | - | | 0.4484 |
| *N1 vs. N2+ N3* |  |  |  |  |  | |  |
| N1 | 22 (4) | 0.85 | 0.79 | NR |  | |  |
| N2+N3 | 39 (12) | 0.82 | 0.71 | 126.5 | 1.81 (0.58, 5.62) | | 0.2976 |
| **Parotidectomy** | | | | | | | |
| Superficial | 47 (10) | 0.88 | 0.78 | NR |  | |  |
| Total | 14 (6) | 0.66 | 0.45 | 38.1 | 0.32 (0.11, 0.92) | | **0.0256** |
| **Neck surgery** | | | | | | | |
| Selective+extended | 13 (2) | 0.92 | 0.77 | NR |  | |  |
| Modified radical | 13 (4) | 0.75 | 0.66 | NR | 2.90 (0.52, 16.31) | |  |
| Radical | 27 (7) | 0.91 | 0.82 | 126.5 | 1.29 (0.27, 6.27) | | 0.3473 |
| **Dose for parotid/upper neck** | | | | | | | |
| ≤54 Gy | 30 (8) | 0.81 | 0.76 | NR |  | |  |
| >54 Gy | 25 (8) | 0.86 | 0.60 | NR | 1.30 (0.49, 3.48) | | 0.5974 |
| **Dose for lower neck** | | | | | | | |
| ≤50 Gy | 11 (1) | 0.90 | 0.90 | NR |  | |  |
| >50 Gy | 45 (15) | 0.82 | 0.66 | 126.5 | 4.57 (0.60, 34.99) | | 0.1092 |
| **Duration of RT treatment** | | | | | | | |
| ≤5.3 weeks | 30 (7) | 0.81 | 0.76 | NR |  | |  |
| >5.3 weeks | 26 (9) | 0.87 | 0.64 | 126.5 | 1.42 (0.53, 3.82) | | 0.4859 |
| **Duration between date of surgery of secondary tumour to date of start of radiotherapy** | | | | | | | |
| ≤4.9 weeks | 29 (8) | 0.87 | 0.66 | NR |  | |  |
| >4.9 weeks | 26 (8) | 0.80 | 0.74 | NR | 1.14 (0.42, 3.09) | | 0.7954 |
