# Supplementary material for: Cutaneous squamous cell carcinoma metastatic to parotid - analysis of prognostic factors and treatment outcome
Source: World J Surg Oncol. 2012 Jun 25;10:117. doi: 10.1186/1477-7819-10-117 (PMC3422189; doi:10.1186/1477-7819-10-117)
Supplement: Additional file 2: — A table showing disease-specific survival. (DOC 109 kb) [file 1477-7819-10-117-S2.doc]

|  | No of patients (No of events) | 2-year survival rate | 5-year survival rate | Median survival (months) | HR | Log-rank test P value |
| --- | --- | --- | --- | --- | --- | --- |
| **Primary tumour size** |  |  |  |  |  |  |
| <20mm | 6 (0) | 1.00 | 1.00 | NR |  |  |
| ≥20mm | 5 (1) | 1.00 | 0 | 56.5 | NE | 0.0833 |
| **Patients with metastasis at different sites** |  |  |  |  |  |  |
| Parotid only | 40 (4) | 0.94 | 0.86 | NR |  |  |
| Both parotid and neck | 26 (4) | 0.86 | 0.79 | NR | 1.52 (0.38, 6.08) | 0.5504 |
| **Margin status of parotid (secondary tumour)** |  |  |  |  |  |  |
| *Patients with metastasis at Parotid only, and both parotid and neck* |  |  |  |  |  |  |
| Positive vs negative vs. close |  |  |  |  |  | **0.0348** |
| Negative | 23 (1) | 0.95 | 0.95 | NR |  |  |
| Positive | 21 (6) | 0.83 | 0.58 | NR | 7.80 (0.94, 64.98) |  |
| Close | 15 (1) | 0.93 | 0.93 | NR | 1.60 (0.10, 25.56) |  |
| Positive vs. no positive |  |  |  |  |  |  |
| No positive margins | 38 (2) | 0.94 | 0.94 | NR |  |  |
| Positive margins | 21 (6) | 0.83 | 0.58 | NR | **6.34 (1.27, 31.53)** | **0.0098** |
| Negative vs. Close |  |  |  |  |  |  |
| Negative | 23 (1) | 0.95 | 0.95 | NR |  |  |
| Close | 15 (1) | 0.93 | 0.93 | NR | 1.44 (0.09, 23.10) | 0.7935 |
| Broad front vs. focally |  |  |  |  |  |  |
| Broad front | 9 (2) | 0.88 | 0.66 | NR |  |  |
| Focally | 12 (4) | 0.80 | 0.53 | NR | 1.79 (0.32, 9.82) | 0.4990 |
| **Margin status of neck (secondary tumour)** |  |  |  |  |  |  |
| *Patients with metastasis at both parotid and neck* |  |  |  |  |  |  |
| Positive vs. no positive |  |  |  |  |  |  |
| No positive margins | 18 (2) | 0.86 | 0.86 | NR | . |  |
| Positive margins | 5 (2) | 0.75 | 0.38 | 56.5 | 3.20 (0.45, 22.96) | 0.2215 |
| Broad front vs. focally |  |  |  |  |  |  |
| Broad front | 1 (1) | 1.00 | 0 | NA |  |  |
| Focally | 4 (1) | 0.67 | 0.67 | NR | 0.58 (0.03, 10.25) | 0.7055 |
| **Perineural Invasion** |  |  |  |  |  |  |
| No invasion | 51 (6) | 0.91 | 0.84 | NR |  |  |
| Invasion | 9 (2) | 0.86 | 0.69 | NR | 1.74 (0.35, 8.62) | 0.4937 |
| **Extracapsular extension** |  |  |  |  |  |  |
| No extension | 26 (3) | 0.90 | 0.82 | NR |  |  |
| Extension | 34 (5) | 0.90 | 0.81 | NR | 1.07 (0.26, 4.47) | 0.9284 |

| **O’Brien Staging** |  |  |  |  |  |  |
| --- | --- | --- | --- | --- | --- | --- |
| *P stage ( P1 vs. P2 vs. P3)* |  |  |  |  |  |  |
| P1 | 35 (3) | 0.93 | 0.89 | NR |  |  |
| P2 | 26 (4) | 0.91 | 0.77 | NR | 1.78 (0.40, 7.95) |  |
| P3 | 2 (1) | 0.50 | 0.50 | 13.2 | 6.06 (0.63, 58.61) | 0.2311 |
| *P stage ( P1 vs. P2+ P3)* |  |  |  |  |  |  |
| P1 | 35 (3) | 0.93 | 0.89 | NR |  |  |
| P2+P3 | 28 (5) | 0.88 | 0.75 | NR | 2.07 (0.49, 8.66) | 0.3084 |
| *N stage (N0 vs. N1 vs. N2)* |  |  |  |  |  |  |
| N0 | 40 (4) | 0.94 | 0.86 | NR |  |  |
| N1 | 7 (2) | 0.71 | 0.71 | NR | 2.52 (0.46, 13.76) |  |
| N2 | 19 (2) | 0.93 | 0.83 | NR | 1.09 (0.20, 5.95) | 0.5254 |
| **AJCC staging** |  |  |  |  |  |  |
| *N1 vs. N2 vs. N3* |  |  |  |  |  |  |
| N1 | 22 (3) | 0.90 | 0.84 | NR |  |  |
| N2 | 38 (5) | 0.90 | 0.81 | NR | 1.05 (0.25, 4.40) |  |
| N3 | 1 (0) | 1.00 | 1.00 | NR | - | 0.9051 |
| *N1 vs. N2+ N3* |  |  |  |  |  |  |
| N1 | 22 (3) | 0.90 | 0.84 | NR |  |  |
| N2+N3 | 39 (5) | 0.91 | 0.82 | NR | 1.01 (0.24, 4.23) | 0.9881 |
| **Parotidectomy** |  |  |  |  |  |  |
| Superficial | 47 (5) | 0.95 | 0.85 | NR |  |  |
| Total | 14 (3) | 0.72 | 0.72 | NR | 0.38 (0.09, 1.60) | 0.1692 |
| **Neck surgery** |  |  |  |  |  |  |
| Selective+extended | 13 (1) | 1.00 | 0.83 | NR |  |  |
| Modified radical | 13 (2) | 0.83 | 0.83 | NR | 2.63 (0.23, 29.57) |  |
| Radical | 27 (3) | 0.96 | 0.86 | NR | 1.22 (0.13, 11.69) | 0.6245 |
